# Supplementary material for: Ancient MAPK ERK7 is regulated by an unusual inhibitory scaffold required for Toxoplasma apical complex biogenesis
Source: Proc Natl Acad Sci U S A. 2020 May 14;117(22):12164–73. doi: 10.1073/pnas.1921245117 (PMC7275706; doi:10.1073/pnas.1921245117)
Supplement: Supplementary File [file pnas.1921245117.sapp.pdf]

PNAS
www.pnas.org

Supplementary Information for

Ancient MAPK ERK7 is regulated by an unusual inhibitory scaffold required for Toxoplasma apical complex biogenesis

Peter S. Back†, William J. O'Shaughnessy†, Andy S. Moon, Pravin S. Dewangan, Xiaoyu Hu, Jihui Sha, James A. Wohlschlegel, Peter J. Bradley*, Michael L. Reese*

Address correspondence to Michael L. Reese or to Peter Bradley
Email: michael.reese@utsouthwestern.edu
Email: pbradley@ucla.edu

This PDF file includes:

Figs. S1 to S7
Tables S1

Other supplementary materials for this manuscript include the following:

AC9 proximity biotinylation proteomics dataset (Dataset S1)
Primers list (Dataset S2)

www.pnas.org/cgi/doi/10.1073/pnas.1921245117

Supplemental Figures

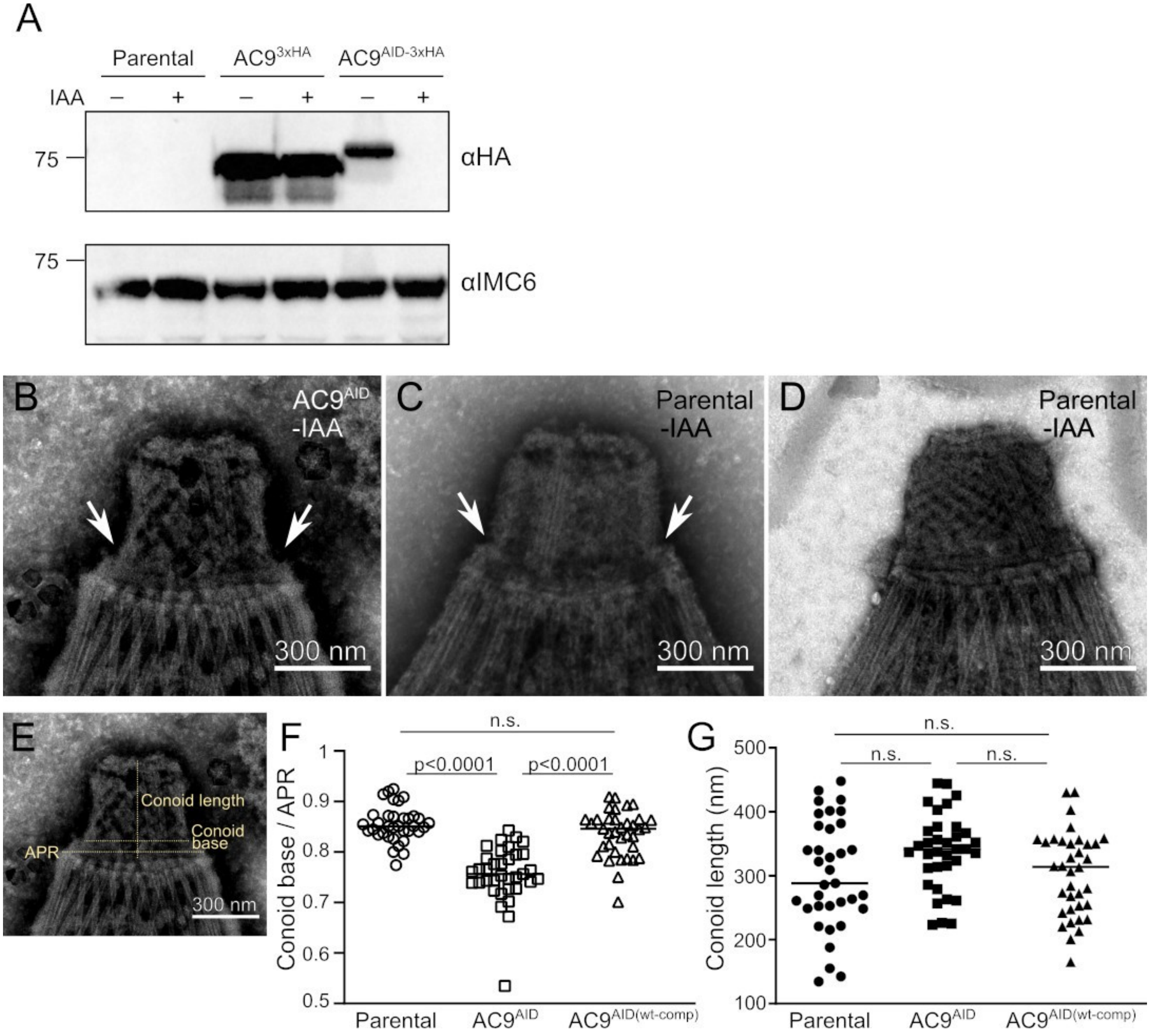

Figure S1. (A) IAA-independent reduction in AC9 protein levels upon AID tagging. Lysates from equal numbers of extracellular parasites of the indicated strains were separated by SDS-PAGE and probed with either anti-HA or anti-IMC6 (loading control). Quantification of band intensities indicates $AC9^{AID\text{-}3xHA}$ levels are 39% of $AC9^{3xHA}$ even when grown without IAA. Western blot also verifies undetectable levels of AC9 after overnight grown in IAA. (B) $AC9^{AID}$ tagging (in the absence of IAA) exacerbates an ultrastructural artifact in TEM sample preparation (arrows) where conoid base slightly detaches from apical polar ring (APR). Note that similar preparations of parental parasites exhibit a similar phenotype (C), though most show stable conoids (D). This observation led us to quantify n=35 conoid TEMs from 2 independent sample preparations for each strain using the rubric in (E); note image is identical to (B). (F) There is a slight, but significant reduction in the ratio of the width of the conoid base over the APR width in AC^{AID} parasites as compared with parental and rescue strains. (G) We observed no significant difference in conoid extension lengths between the three strains. Significance estimated by 1-way ANOVA with Tukey's test.

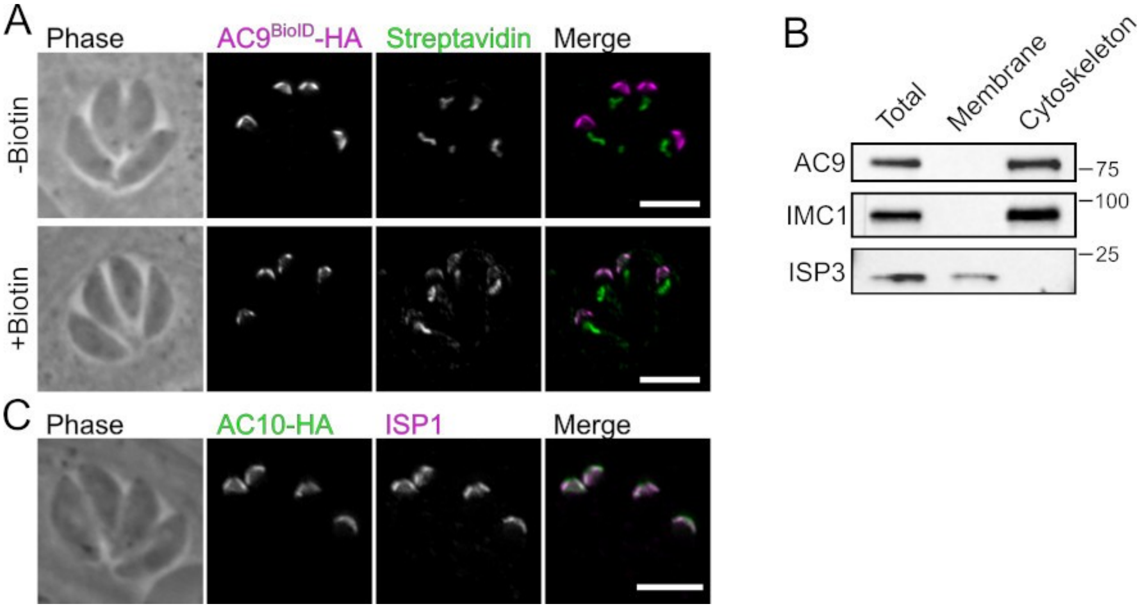

Figure S2. Preparation of apical cap cytoskeleton for BioID. (A) $AC9^{BioID}$-3xHA (magenta) actively biotinylates proteins at the apical cap when parasites are grown in the presence of 150 μM biotin, as detected by streptavidin staining (green). Note that the parasite apicoplast contains natively biotinylated proteins that are recognized by streptavidin. (B) Cytoskeletal components of the apical cap such as AC9 and IMC1 are enriched by detergent fractionation, while membrane-associated apical cap proteins, such as ISP3, are de-enriched. (C) Of the top hits in our BioID dataset (Supplemental Data 1) was one previously unidentified apical cap protein, TGGT1_292950, which, when endogenously tagged at its C-terminus with 3xHA (green) was verified to localize to the apical cap (ISP1; magenta). All scale bars: 5 μm.

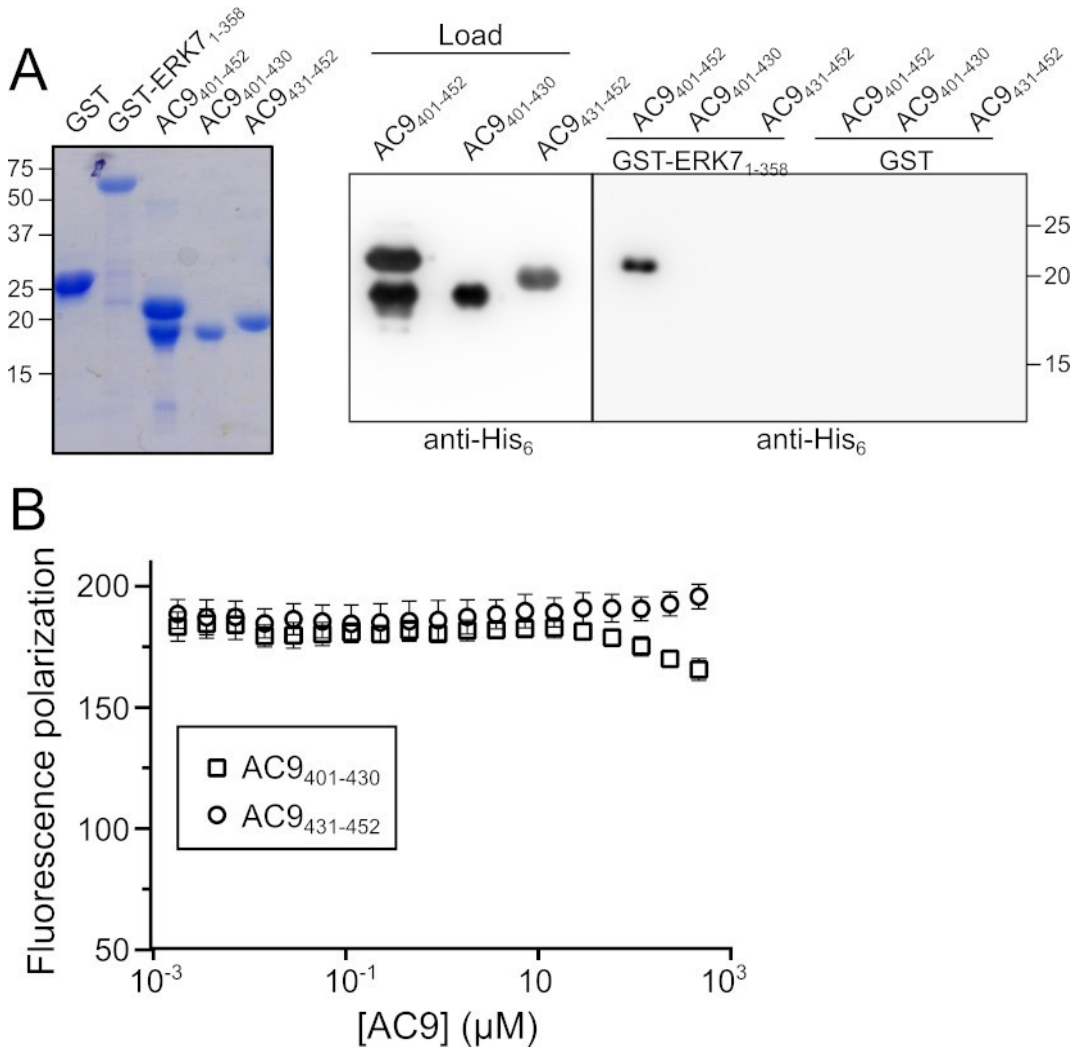

Figure S3. AC9 binds the ERK7 kinase domain. (A) (Left panel) Coomassie stained gel of purified proteins used to test binding. (Right panel) His_6-SUMO fusions of the indicated AC9 fragments were incubated with GST or GST-ERK7 bound to glutathione sepharose resin, washed, and detected by western blot with anti-His_6 antibody. Only $AC9_{401-452}$ showed detectable binding to ERK7. (B) Fluorescence polarization competition in which $AC9_{401-430}$ and $AC9_{431-452}$ were titrated against fluorescein-labeled $AC9_{419-452}$ bound to TgERK7. Note that neither the N-terminal nor C-terminal fragments of the AC9 ERK7-binding region were able to efficiently compete with $AC9_{419-452}$ for binding, indicating the fragments have affinities >1 mM for the kinase.

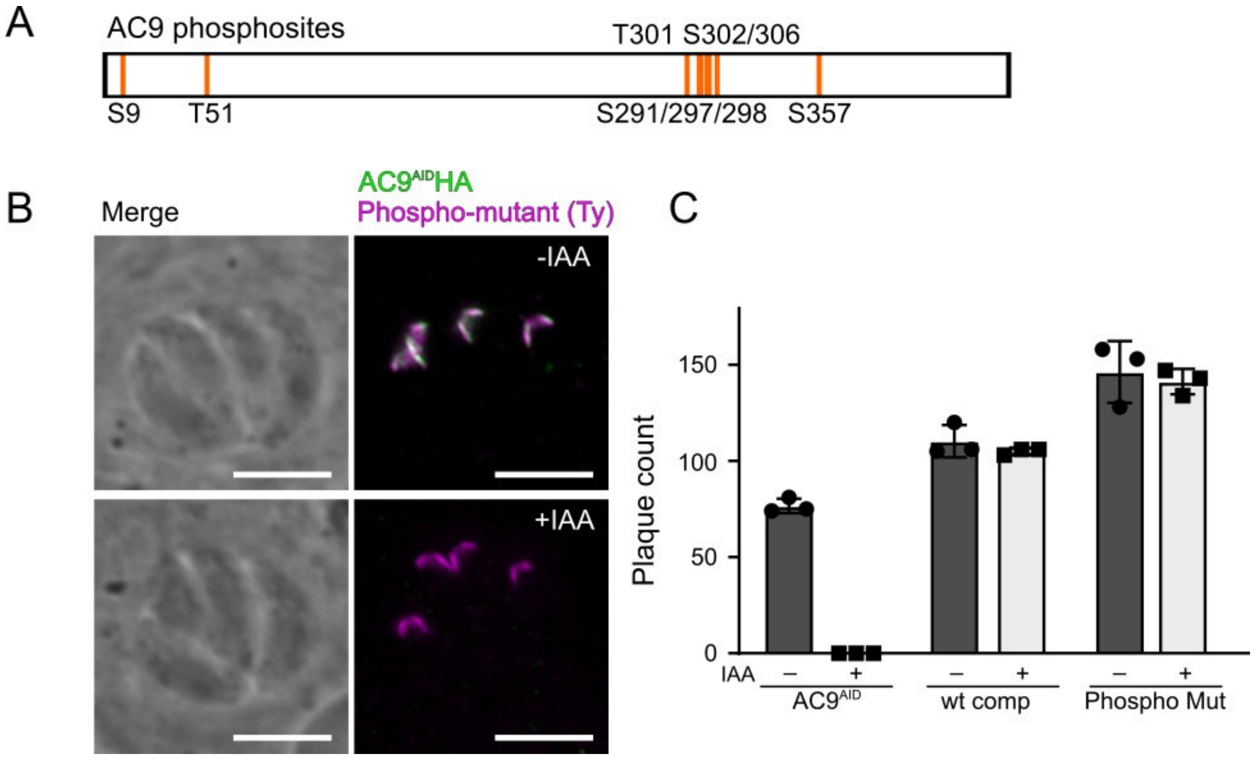

Figure S4. Phosphorylation of AC9 is not required for its function. (A) Location of known phosphorylated Ser/Thr on AC9 that have been mutated to Ala in this study. (B) Phospho-mutant AC9 correctly localizes to the apical cap in the AC9^{AID} background. Scale bars: 5 μm. (C) The phospho-mutant is able to fully rescue plaque formation in the AC9^{AID} background, indistinguishably from complementation with a wild-type copy of AC9.

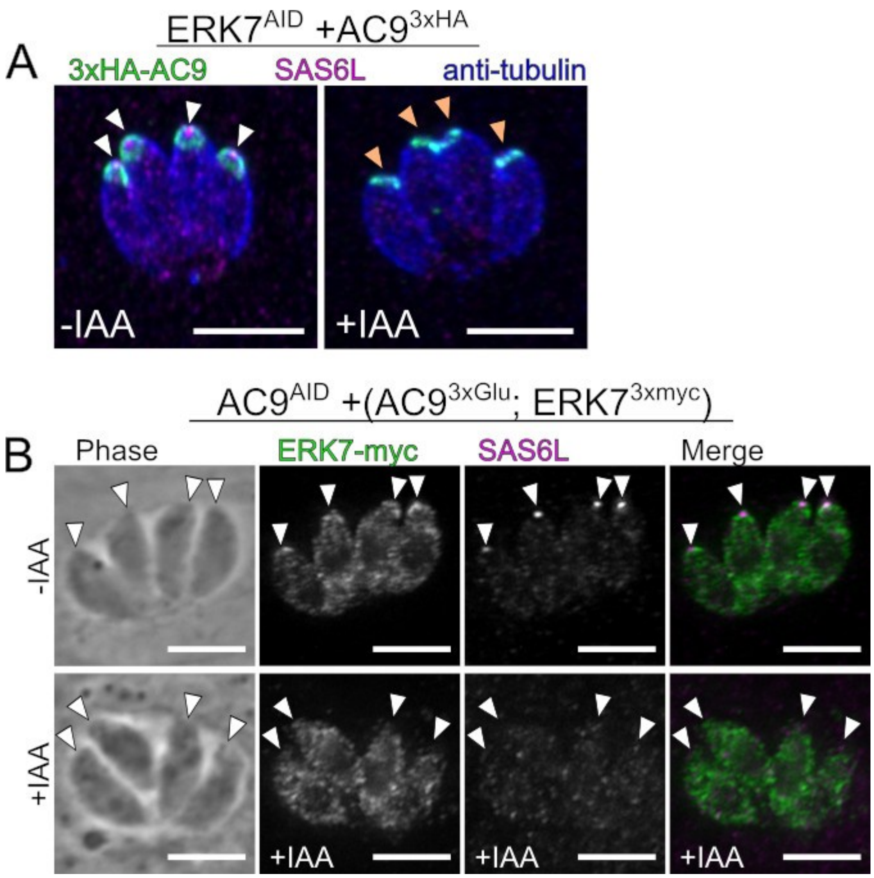

Figure S5. (A) AC9 localization is unaffected by ERK7 degradation. ERK7^{AID}(3xHA-AC9) parasites were grown in the presence or absence of IAA and stained with anti-HA (AC9; green), anti-SAS6L (magenta), and anti-β-tubulin (blue). Note anti-β-tubulin does not stain conoid, likely due to antigen accessibility. Images are maximum intensity projects of confocal stacks. White arrowheads indicate SAS6L-positive conoid puncta. Orange arrowheads indicate expected localization of missing SAS6L puncta in ERK7$^{AID/IAA}$ parasites. (B) AC9^{AID}(+AC9^{3xGlu}; ERK7-3xmyc) parasites were grown in the presence or absence of IAA and stained with anti-myc (ERK7; green) and anti-SAS6L (magenta), a marker for the parasite conoid. Arrows indicate the parasite apical end. Note the bright SAS6L foci in the -IAA parasites that are lost in +IAA. All scale bars: 5 μm.

Data collection		Refinement	
**Wavelength**	1.541	**Reflections used in refinement**	39043 (3083)
**Resolution range**	29.89 - 2.1 (2.175 - 2.1)	**Reflections used for R-free**	1718 (141)
**Space group**	P 1	**R-work**	0.1583 (0.1787)
**Unit cell**	50.8897 52.9379 67.8003 82.55 84.5785 81.35	**R-free**	0.2119 (0.2329)
**Total reflections**	93549 (4051)	**CC(work)**	0.961 (0.919)
**Unique reflections**	39049 (3083)	**CC(free)**	0.924 (0.809)
**Multiplicity**	2.4 (1.3)	**Number of non-hydrogen atoms**	5986
**Completeness (%)**	96.68 (76.39)	**macromolecules**	5468
**Mean I/sigma(I)**	15.17 (3.48)	**ligands**	20
**Wilson B-factor**	17.81	**solvent**	498
**R-merge**	0.05342 (0.1976)	**Protein residues**	682
**R-meas**	0.0665 (0.2744)	**RMS(bonds)**	0.004
**R-pim**	0.03903 (0.1898)	**RMS(angles)**	0.61
**CC1/2**	0.997 (0.894)	**Ramachandran favored (%)**	98.33
**CC***	0.999 (0.972)	**Ramachandran allowed (%)**	1.67
		**Ramachandran outliers (%)**	0.00
		**Rotamer outliers (%)**	0.17
		**Clashscore**	1.45
		**Average B-factor**	29.43
		macromolecules	29.18
		ligands	44.08
		solvent	31.59
		Number of TLS groups	16

Table S1. Data collection and refinement statistics. Statistics for the highest-resolution shell are shown in parentheses.

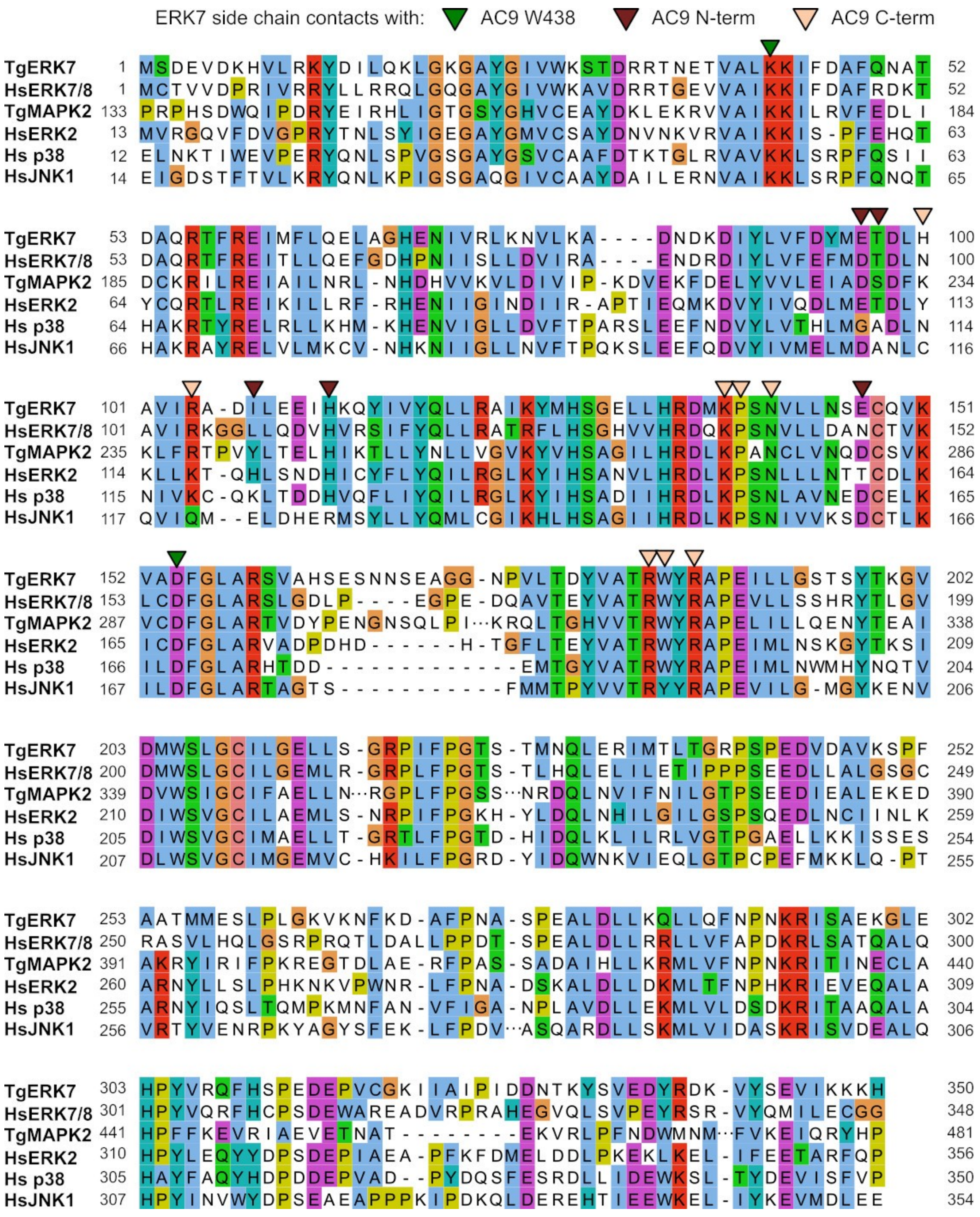

Figure S6. Alignment of TgERK7 and TgMAPK2 kinase domains with human MAPKs. TgERK7 side chains that make contact with $AC9_{419\text{-}452}$ in our crystal structure are marked as indicated. Large gaps were removed from alignment and are indicated with ellipses.

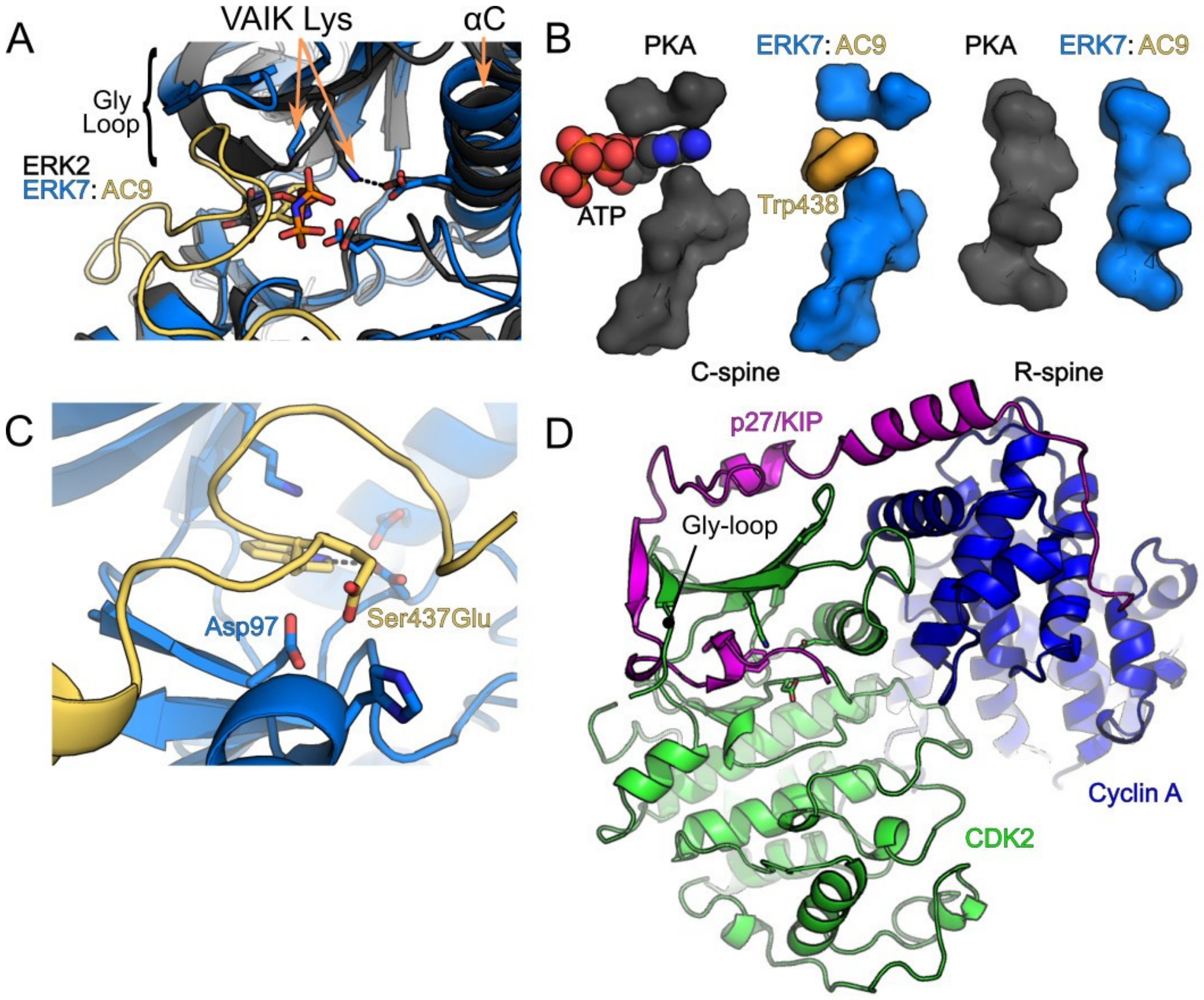

Figure S7. Comparison of ERK7:AC9 with other kinase structures. (A) The structure of activated, AMP-PNP-bound ERK2 (black; 6OPG) is overlaid with ERK7:AC9 (blue and yellow) and the Gly-loop, catalytic lysine, and αC helices are indicated. Note that the ERK7:AC9 Gly-loop is held in an inactive conformation by AC9, which keeps the αC-Glu from salt bridging with the VAIK Lys.(B) The "C-spines" (PKA: V57, A70, M128, L172, L173, I174, L227, M231; TgERK7: V27, A40, L99, V142, L143, L144, I210, L214) and "R-spines" (PKA: L95, L106, Y164, F185; TgERK7: L64, L76, H134, F155) of activated, ATP-bound PKA (black; 1ATP) is shown in comparison to ERK7:AC9 (blue and yellow). While the R-spine is intact, the C-spine is not fully complete by Trp438; ERK7 is not in an active conformation when bound to AC9, as the Gly-loop is in an open conformation, and the VAIK:α-C Glu salt bridge are not formed (see (A) and Figure 5). In addition, AC9 displaces the ERK7 activation loop (see Figure 5D,E). (C) Mutation of AC9 Ser437 to Glu would clash with the side chain of Asp98 (<3.5 Å between the carboxylate side chains) when AC9 is in the optimal conformation for binding ERK7. (D) Overview of the CDK2:p27/KIP:Cyclin inhibitory complex (1JSU). p27 (magenta) wraps around the cyclin A (blue) and CDK2 (green), unfolding the CDK2 Gly-loop and inserting it's C-terminal residues into the active site, replacing nucleotide.
